# Evaluation of different types of enrichment - their usage and effect on home cage behavior in female mice

**DOI:** 10.1371/journal.pone.0261876

**Published:** 2021-12-23

**Authors:** Ute Hobbiesiefken, Paul Mieske, Lars Lewejohann, Kai Diederich

**Affiliations:** 1 German Federal Institute for Risk Assessment (BfR), German Center for the Protection of Laboratory Animals (Bf3R), Berlin, Germany; 2 Institute of Animal Welfare, Animal Behavior and Laboratory Animal Science, Freie Universität Berlin, Berlin, Germany; Istituto Superiore Di Sanita, ITALY

## Abstract

Numerous studies ascertained positive effects of enriched environments on the well-being of laboratory animals including behavioral, physiological and neurochemical parameters. Conversely, such conclusions imply impaired animal welfare and health in barren husbandry conditions. Moreover, inappropriate housing of laboratory animals may deteriorate the quality of scientific data. Recommendations for housing laboratory animals stipulate that cages should be enriched to mitigate adverse effects of barren housing. In this context, it is not only unclear what exactly is meant by enrichment, but also how the animals themselves interact with the various items on offer. Focal animal observation of female C57BL/6J mice either housed in conventional (CON) or enriched (ENR) conditions served to analyze the impact of enriching housing on welfare related behavior patterns including stereotypical, maintenance, active social, and inactive behaviors. CON conditions resembled current usual housing of laboratory mice, whereas ENR mice received varying enrichment items including foraging, housing and structural elements, and a running disc. Active and inactive use of these elements was quantitatively assessed. CON mice showed significantly more inactive and stereotypical behavior than ENR mice. ENR mice frequently engaged with all enrichment elements, whereby riddles to obtain food reward and the running disc preferably served for active interactions. Offering a second level resulted in high active and inactive interactions. Structural elements fixed at the cagetop were least attractive for the mice. Overall, the presented data underline the positive welfare benefits of enrichment and that mice clearly differentiate between distinct enrichment types, demonstrating that the perspective of the animals themselves should also be taken into account when specifying laboratory housing conditions. This is particularly important, as the ensuring of animal welfare is an essential prerequisite for reliable, reproducible, and scientifically meaningful results.

## Introduction

Refinement, i.e., reducing pain and distress for those animals in animal based research that cannot be replaced or reduced in numbers, is one of the major goals of the 3R principle of Russell and Burch [1]. Over the recent years, the term "refinement" was broadened to methods that enhance animal welfare [2]. Therefore, when using the term "refinement", most scientists are essentially talking about improving the experimental as well as the living conditions of laboratory animals in a way that is aimed at improving animal welfare. Our aim is to go beyond fulfilling the measures formulated as the five freedoms [3] to providing the necessities for a life worth living [4]. It is of note that current definitions of animal welfare emphasize that the status of the subjectively perceived quality of life of an individual is a core feature of animal welfare [5]. Thus, refinement should not only look good to us, but must also be perceived as an improvement by the animals themselves, thus creating a bridge between observed events such as behavior and physiological parameters and subjective perception from the animal’s point of view [6].

Furthermore, this definition of animal welfare emphasizes that welfare is measured on more than one scale. This means that different welfare indicators interact and should be considered for an overall view. For example, the compromised welfare of a slightly injured animal can be enhanced by providing good feeding condition and sociopositive interactions at the same time. Therefore, also refined housing has a compensatory potential for other treatments that may not be as easily refined in animal studies.

Recent advances in laboratory animal science and modern legislation support the holistic refinement approach. For example, the EU Directive 2010/63 stipulates that animals should be provided with ‘space of sufficient complexity to allow expression of a wide range of normal behavior’, aiming to overcome behavioral deprivation caused by impoverished housing conditions [7]. Such formulations are vague and thus tend to describe the minimum standard. This standard usually implies providing laboratory mice with bedding material, nesting material and a shelter. As a side note, nesting and shelter are additions that 20 years ago were themselves described as enrichment compared to cages filled only with bedding [8]. For the purposes of this paper, we like to assume that shelter and nesting materials are now indeed considered to be the usual.

In fact, historically, any additions intended to raise the complexity of impoverished conventional housing has been considered as enrichment. By this "enrichment" has become an umbrella term for a variety of shelters, bedding and nesting materials, and various objects, or any combination thereof, and lacks a general theoretical framework of what should be considered enrichment [9].

Despite the ambiguity that therefore remains to be considered for the terminology, one can essentially find a number of positive effects on the well-being of laboratory mice through any refinement of the husbandry conditions. Regarding the influence on behavior, enriching the environment of mice led to a decreased expression of abnormal repetitive behaviors, i.e., stereotypies [10–13], is known to decrease behavioral measures of anxiety [10, 14, 15] and reduced the development of a depressive-like phenotype in mice [16]. Furthermore, enriching the housing conditions of mice led to improved memory function and learning abilities [17, 18] accompanied by an increase of hippocampal neurogenesis [15, 18, 19]. Growth and stress physiology [10, 20] was as well seen to be positively influenced in mice in enriched housing conditions compared to conventional housing conditions. Access to enrichment also mitigated stress responses and supported natural killer cell activity in mice as important well-being parameters [14]. Studies with rats have also shown a major influence of housing conditions on the affective state and cognitive bias, thus also the emotional well-being of laboratory animals [21]. In the context of the reproducibility crisis, it is worth noting that concerns that enrichment in general increases the variation in experimental results have not been confirmed [10, 20, 22–25].

Summarizing the current literature, it can be taken as a fact that providing a more diverse living environment has a major impact on the well-being of laboratory animals. Indeed, most current recommendations for housing laboratory mice include the advice to provide environmental enrichment [5, 20, 26]. However, this has not yet led to a widespread implementation of any of these measures. Moreover, since various types of enrichment have been used over the recent years, it is not surprising that there is still no consensus on the most suitable types of enrichment for enhancing animal welfare, as they are based more on anthropomorphic feelings and convenience [9].

We therefore urgently need a strategy for an appropriate assessment of cage design. Enrichment can already be seen as an improvement with regard to the question of animal health, yet the second question demanded by Dawkins [27], what the animals themselves want in order to be able to see it as a real improvement in animal welfare, has so far remained largely unanswered.

In order to evaluate the suitability of enrichment items for enhancing animal welfare of laboratory animals, different approaches are feasible. Of special importance are animal centric strategies [28] to investigate how different items are perceived by the animals themselves [5, 29, 30]. A very promising approach is to "ask" the animals by performing home cage-based preference studies [31]. However, knowing that mice prefer an enriched environment to more barren conditions alone is not revealing the perceived importance of specific enrichment items. To do this, it is necessary to conduct a series of tests comparing preferences for different items, as we have accomplished in an article that is published in parallel [32]. In order to find out how the animals actually interact with the enrichment items, detailed direct behavioral observations are necessary. This is, however, laborious and time consuming and so far only little data has been published in that regard [22]. Here we observed a wide range of home cage behavior with and without enrichment items being present. Mice living in conventional laboratory housing conditions (bedding and nesting material plus a shelter) served as a control group. Different types of enrichment are likely to induce different ways of how the animals can interact. For example, a running wheel is essentially different from a plastic tube or a wooden angle although all items fall under the umbrella term enrichment. To address this lack of specificity we suggest a categorization according to the primary use to distinguish between different classes/categories of enrichment, namely cage structuring and climbing objects (structural enrichment), puzzles for active foraging engagement (foraging enrichment) and alternative shelters (housing enrichment). In direct observations, we analyzed the interaction with these items and thereby could assess how the animals themselves might perceive the enrichment.

## Material and methods

### Ethics statement

All experiments were approved by the Berlin state authority, Landesamt für Gesundheit und Soziales, under license No. G 0069/18 and were in accordance with the German Animal Protection Law (TierSchG, TierSchVersV). The study was preregistered in the Animal Study Registry (ASR, DOI 10.17590/asr.0000062).

### Animals and housing condition

The 24 female C57BL/6J mice used in this study were obtained from a commercial breeder (Charles River Laboratories, Sulzfeld, Germany) at an average age of 8 weeks. Mice were randomly allocated to groups of four animals in Makrolon type III cages (L x W x H: 598 x 380 x 200 mm, Tecniplast, Italy) by a researcher not involved in the experiment; animals were alternately assigned to the groups (enriched and conventional) to avoid bias. Conventional housing contained aspen bedding material (Polar Granulate 2–3 MM, Altromin), paper (cellulose paper unbleached 20x20 cm, Lohmann & Rauscher International GmbH & CO KG), cotton roll nesting material (dental cotton roll size 3, MED-COMFORT), a 15 cm acrylic glass tunnel (Ø 4cm PMMA xt®, Gehr®) and a red triangle plastic house (mouse house, TECNIPLAST®). Regular rodent food (autoclaved pellet diet, LAS QCDiet, Rod 16, Lasvendi, Germany) and tap water *ad libitum* were provided. The climate was maintained at a room temperature of 22°C (+/- 2), room humidity at 55% (+/- 15) and a 12/12 light/dark cycle regimen (lights off: 8 pm) with an automatically simulated 30 min sunrise in the morning using a wake-up light (HF3510, Philips, Germany). Mice were looked after daily and during weekly cage cleaning a more detailed visual health check was performed. For avoidance of stressful handling and implementing refinement methods, all animals were trained to tunnel handling [33] daily during an habituation phase of 3 weeks (for a detailed protocol see: [34]). Tunnel handling was used whenever the handling of the mice was needed during weekly cage cleaning and experiments.

At 11 weeks of age, the mice were transferred to the respective housing condition (conventional, n = 12; enriched, n = 12) and remained in their familiar social group of four mice.

The conventional housing group remained in their previous housing conditions but the acrylic glass tunnel that was used for habituation of tunnel handling was removed. Mice in the enriched housing groups received a running wheel with mouse igloo, paper nesting, and cotton rolls as permanently provided enrichment items (Table in S4 Table). In addition five weekly changing and randomly selected enrichment items from the categories "structural", "housing", "nesting", and "foraging " (Table in S5 Table) were provided. Change of the enrichment took place during the weekly cage cleaning process. The enrichment elements were randomized before the start of the husbandry period. The cages were then enriched and weekly changed according to this protocol. Structural elements hereby should serve as an element for exploration and resting purposes, housing elements for resting during the inactive phase and climbing throughout the active phases. Items from the category of "nesting" contained different nesting materials and the elements categorized as "foraging " contained different riddles for cognitive stimulation of the animals. As a reward for solving the riddles, the foraging elements were filled daily with a small amount (3.5 g) of millet seed (organic peeled golden millet, Spreewälder Hirsemühle or Bohlsener Mühle). 3D printing templates of two riddles can be found in the (files in S1 File, S2 File). Mice of the conventional housing group received the same amount of millet seed presented as a pile on the bedding material. Prior to the home cage video observations, the mice were used in other experiments including indirect calorimetry and x-ray bone density measurement but remained in their respective housing conditions. The results of these investigations are not part of this manuscript. During the housing period and before the start of the first experimental observation, one mouse of the conventional housing condition had to be excluded due to congenital health problems not related to the experimental conditions. In total, the behavior of 11 conventional kept mice and 12 enriched kept mice was recorded. After completion of the experiments in this work, the animals remained in their housing conditions and were used for further studies.

### Animal identification

At the age of 9–10 weeks, all mice received a microchip transponder (ISO 11784/85, FDX-B transponders, Planet ID®) for individual identification. It was placed under the skin of the dorsal neck region in rostrocaudal implantation direction under general isoflurane anesthesia and pain relieve (Metacam ®, Boehringer Ingelheim (Schweiz) GmbH). Pain medication was also administered orally the following three days after implantation. For direct optical identification, all mice were color-coded once a week on the base of the tail using permanent markers (Edding® 750).

### Body weight

Body weights of the mice of the conventional (n = 11) and enriched (n = 12) housing group were measured before the start of the first experiment after a housing period of 16 weeks in the respective conditions. Using tunnel handling, the mice were weighed in a weighing pan on a standard scale (Kern® EMB 200–2).

### Experiment 1: Observation of homecage behavior

Home cage video observations of the enriched and conventional housed mice took place after 16 weeks of housing in the different housing conditions. Videos were recorded on three consecutive days. Two groups were filmed each day in a randomized order from 19:00 to 07:00 o´clock the following day under dim red- and IR- light (LED bar lights SBL-0140-RD, MBJ imaging GmbH; LED spot outdoor IR spotlight, Synergy21 ALLNET GmbH). An infrared camera (Basler USB [acA1920 – 40um]) was placed in front of the long side of the cage and connected to a laptop with recording software (iSpy Video Surveillance Software [v7.2.0.0]).

### Experiment 2: Observation of enrichment use

After a housing period of 29 weeks in the enriched cages, home cage video observation of five randomly assigned enrichment combinations was conducted for the evaluation of the use of the presented items. During the housing period, all enrichment items were presented for extensive habituation. All 12 mice from the enriched housing group were included in this video observation.

The observations took place once a week (five weeks in succession). The enrichment items consisted of the permanently provided items (running wheel with mouse igloo, paper nesting, rolled cotton wool) and five weekly changing randomized items from "structural", "housing", "nesting", and "foraging" categories.

Between 16:30 and 8:00, home cage behavior was recorded under dim red- and IR- light conditions. A second video recording was conducted the following day between 8:00 and 9:20 directly after filling the foraging enrichment items with millet seed to analyze the use of these interactive items.

**Fig 1:**
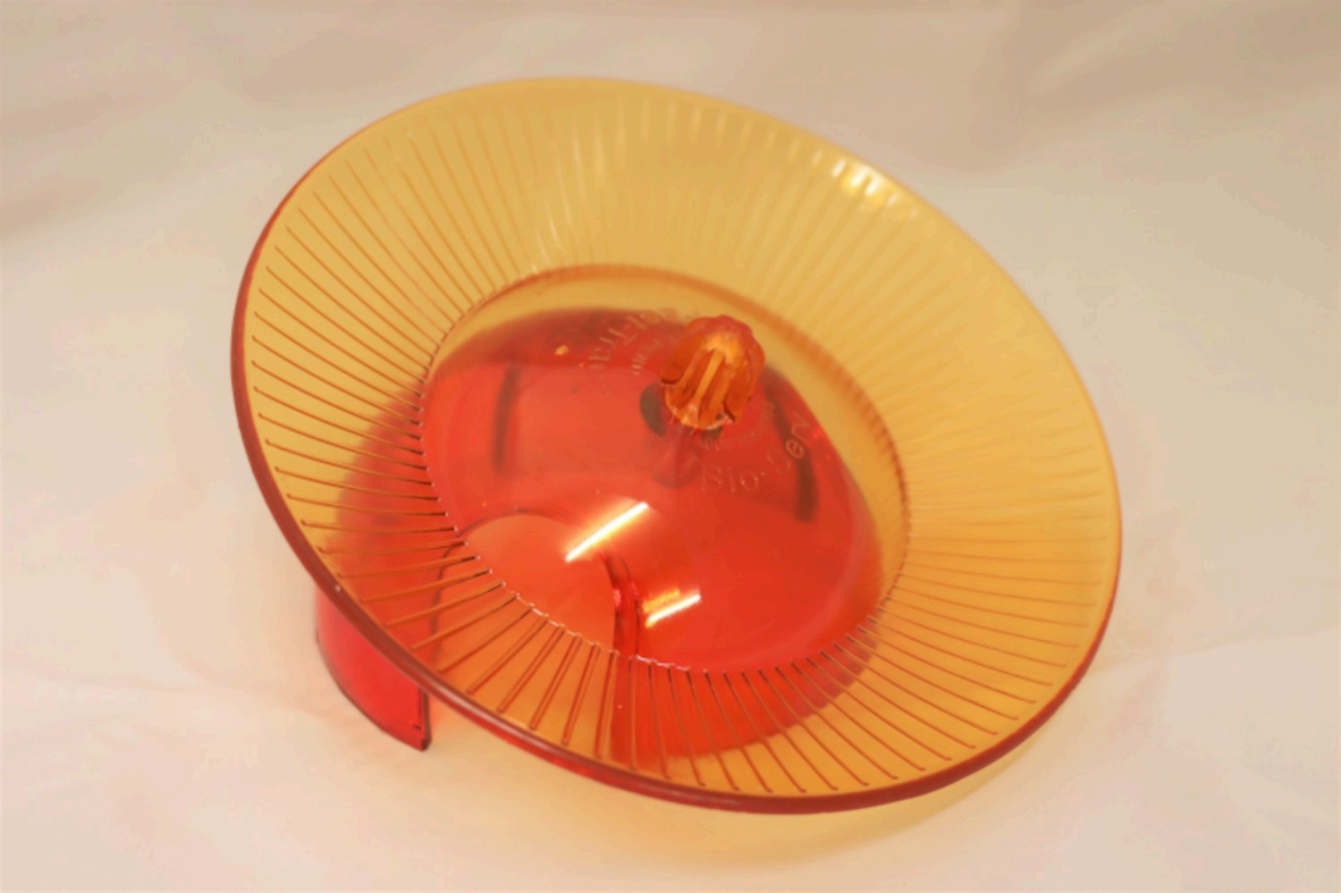
Running disc.

**Fig 2:**
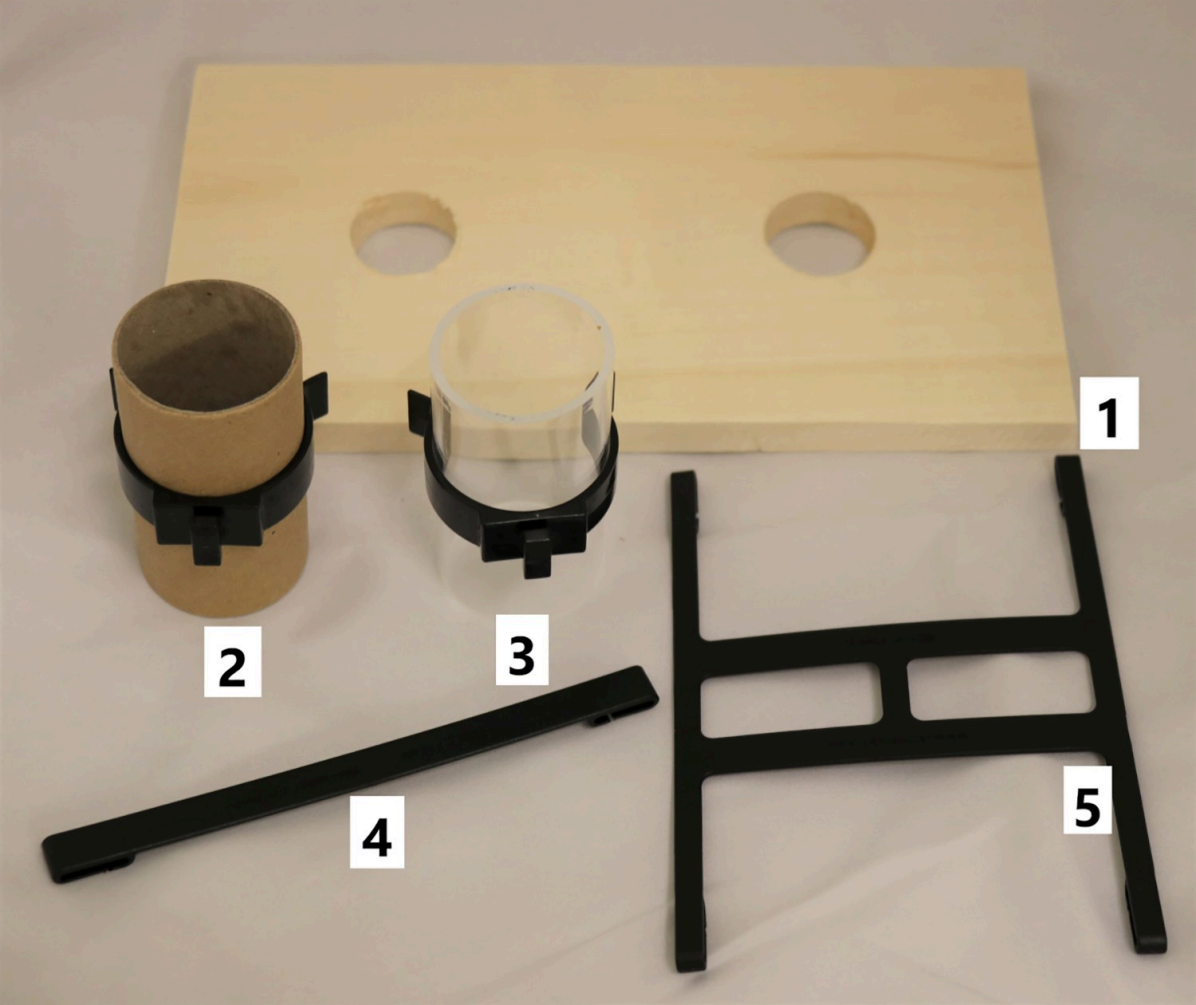
Enrichment items from the category structural. 1 second level 2 holes, 2 clip with paper tube, 3 clip with plastic tube, 4 mouse swing, 5 mouse swing double.

**Fig 3:**
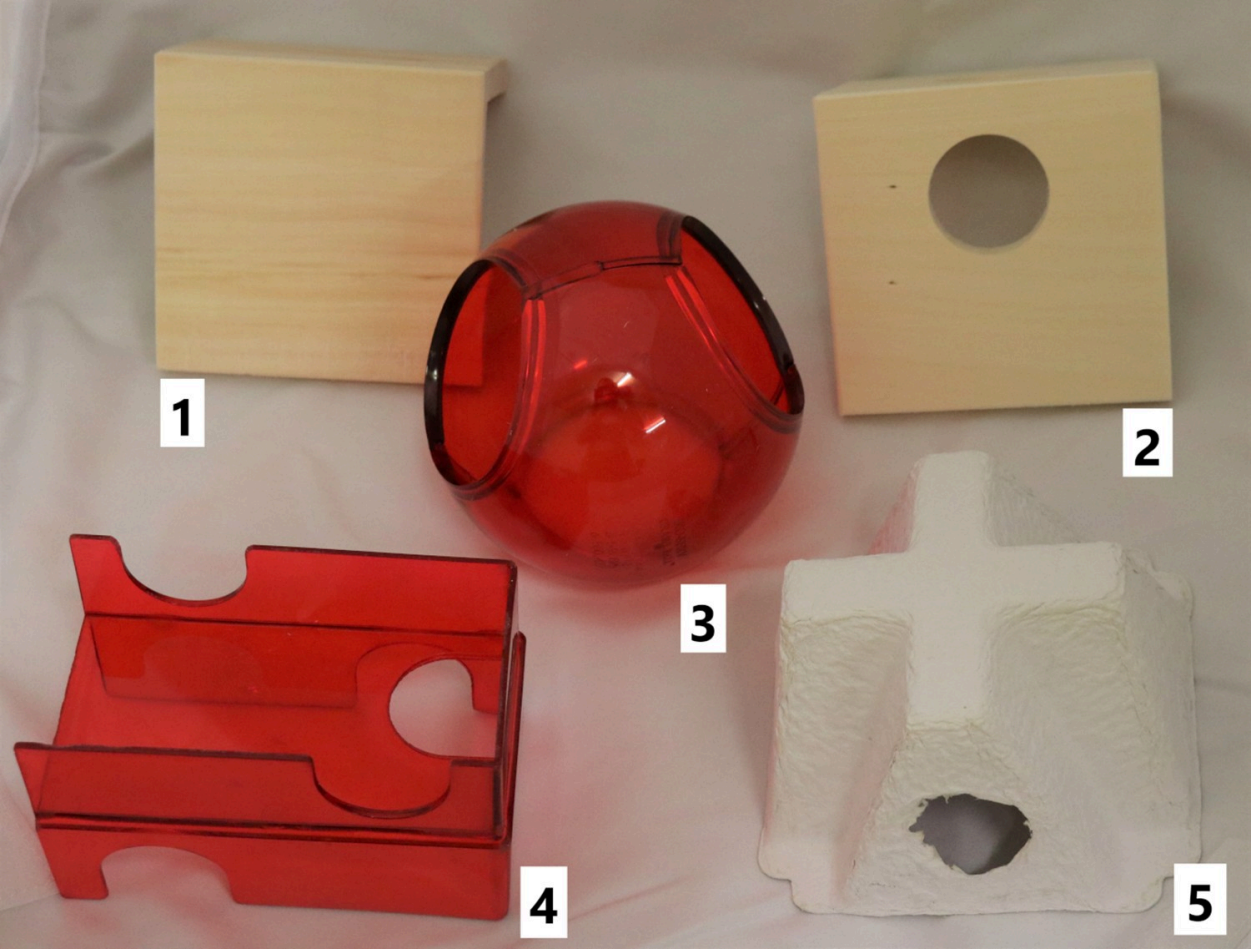
Enrichment items from the category housing. 1 wooden angle, 2 wooden angle with hole, 3 house ball 4 floor house 5 paper house.

**Fig 4:**
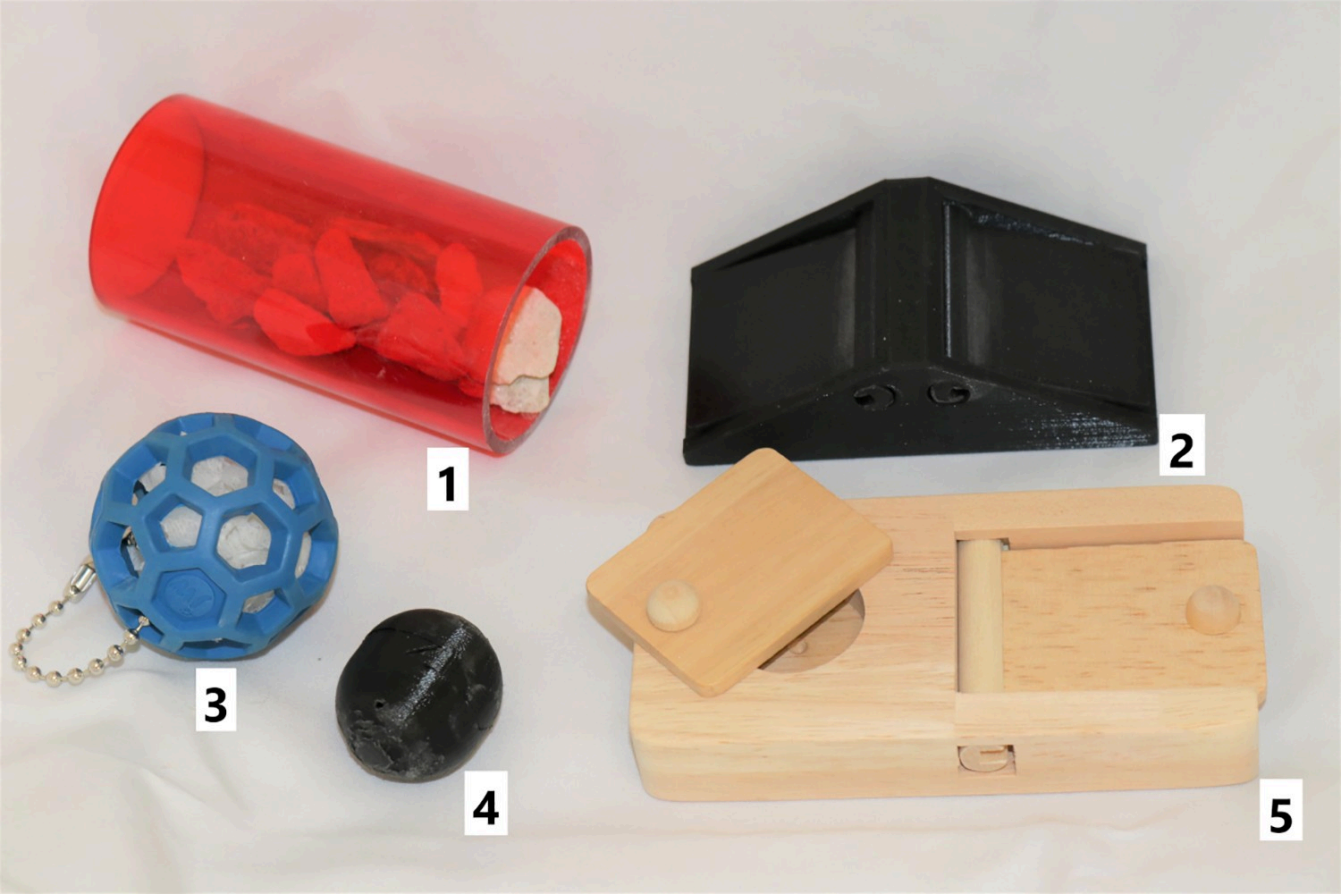
Enrichment items from the category foraging. 1 tube with stones, 2 flap puzzle, 3 lattice ball, 4 treat ball, 5 sliding puzzle.

Through this procedure, five different constellations of enrichment were observed. The dark phase observation served for analyzing the use of the running disc (Fig 1), structural (Fig 2) and housing (Fig 3) elements. The light phase observation served for analysis of the foraging enrichment elements (Fig 4), as the mice were habituated to receive their millet reward during this time period and were therefore active in this period. 3D Printing templates of the flap puzzle and treat ball can be found in the (S1 and S2 Files). The analysis of the usage of the foraging items was separated from the other categories in order to prevent biased data due to the feeding regime.

### Analysis

#### Experiment 1: Observation of home cage behavior

The behavior of each animal was analyzed in the first 15 min of the second (8 pm), third (9 pm), fourth (10 pm) and fifth (11 pm) hour of the dark phase using the one-zero sampling method with 15 sec intervals and focal animal sampling [35]. Video processing was done with computer software (ffmpeg v 4.2.2) resulting in 60 minutes video observation per cage processed in 240 datapoints per mice. Videos were then analyzed by one observer using an adapted ethogram (Table 1) in accordance with established ethograms of mice [36–38]. A selected behavior was counted if it was shown over a period of at least three seconds in a 15 second interval or repeated at least three times in succession within the 15 second interval. Behaviors of each mouse were then calculated as mean per 240 datapoints and given in percent.

**Table 1:** Ethogram used for behavioral analysis in experiment 1.

category	behavior	Definition
**stereotypical behavior**	**route tracing**	A mouse will trace out an identical, repeated route on the cage floor or on the cage floor involving the cage lid. At least 3 repetitions.
	**jumping**	Jumping is a repetitious upright motion towards the cage top. Sometimes when rearing, mice may jump up towards the cage lid. At least 3 repetitions.
	**circling**	A circling mouse repetitively traces a loosely circular path hanging on the cage top. While circling, the mouse may change its body orientation, alter its direction, and intermix other behaviors while following the path. At least 3 repetitions.
	**bar- orientated**	Chewing or sniffing at the cage bar for at least 3 seconds or at least 3 times in succession.
	**wiping**	A mouse rears against the side of the cage and sways from side to side. While the hind-legs usually remain stationary, the forepaws and head move in an arc from side to side that may reach down to the floor on either side of the body. At least 3 repetitions.
	**scratching**	A mouse is scratching at the cagewall, usually in one corner of the cage, may be mixes with burrowing. At least 3 repetitions.
**inactive**	**inactive**	Sitting or lying motionless at least 3 sec throughout the recording interval.
**maintenance behavior**	**drinking**	The mouse rears up and licks the nozzle of the drinker.
	**feeding**	The mouse rears up to gnaw at food pellets through the bars of the food hopper or sits on the hind-legs while chewing at a piece of food pellet in its forepaws.
	**self- grooming**	Usually in a sitting position, the mouse will lick its fur, groom with the forepaws, or scratch with any limb.
**active social**	**cagemate-grooming**	A mouse licks the fur or body of another mouse (cagemate).

#### Inter rater reliability

A subset of the videos of 12 randomly chosen mice (6 enriched, 6 conventional) was analyzed by a second observer. We calculated agreement for each of the 11 behaviors in all 240 data samples per mouse. This led to 2640 comparisons per mouse and an overall number of 31,680 samples for each of the two observers. It is of note that this sample is not a fully crossed rating of all observations but we deemed the sample size sufficient to calculate the Cohen´s kappa as a measure of inter rater reliability [39].

#### Experiment 2: Observation of enrichment use

To analyze the use of the enrichment elements, behavior of the first 15 min of the second and third hour of the dark phase was analyzed using one-zero sampling with 15 s intervals and focal animal sampling [35]. The recordings were cut into 15 sec intervals (ffmpeg v 4.2.2) and were then analyzed visually by one observer and stored in an Excel® table (Microsoft® Excel® (Version 2016)). Same procedure and analysis were performed with daytime video observations from 8:00 to 8:15 and 9:00 to 9:15 for the foraging enrichment items, which were then filled with millet seed. The 30 minutes of video recording at daytime led in total to 120 datapoints per mouse and observation. Please note that observing behavior in group housing comes with a statistical caveat. The individual use of enrichment items within the same housing unit might not be independent of the use of the same items by cage mates. However, the one-zero sampling method with intervals of 15 seconds is considered a reasonable time window for every mouse to be able to interact independently with the desired items. By accumulating 120 independent observations, we were able to obtain a good overview of the actual interaction with the enrichment items. Enrichment items and the definition of their active and/or inactive use are listed in an ethogram (Table in S6 Table). Because the use of the different nesting materials could not be analyzed with sufficient precision, we excluded this category from the analysis. For all remaining enrichment items, the active use was defined as sitting on, running through/over, gnawing at, sniffing at, or manipulating the item. Inactive use was defined as sitting/resting/grooming in/under the item or sleeping on/inside. Active and inactive use of each item was then calculated as mean usage per 120 data points and displayed as percent.

### Statistics

#### Body weight

Body weights were analyzed using R (version 4.0.4., R Studio version 1.3.959). The Shapiro-Wilk test determined a normal distribution (p>0.05) for the weight data of the conventional (n = 11) and enriched (n = 12) housed group. The homogeneity of the variances could be confirmed by means of F-test (p>0.05), whereupon a t-test was performed.

#### Experiment 1 and 2

For the observation of the home cage behavior, exploratory statistical analyses were performed using R (version 4.0.4., R Studio version 1.3.959). The Shapiro-Wilk test determined non-normal distribution of the data (p < 0.05). The Wilcoxon-Rank-Sum test was used to compare behavioral data between conventional and enriched housing. A value of p < 0.05 was considered to represent a statistically significant difference. Data are presented either as boxplots (Fig 6) showing the median and interquartile range from 25th to 75th percentile or mean and standard deviation in the results text.

The data of the enrichment use, the mean percentage of active and inactive use of the items was calculated and presented as a stacked barplot with Microsoft® Exel® (Version 2103). Due to the individual optical tailmarks of each animal and the obvious enrichment items in the enriched housing group, the observer could not be blinded to the treatment condition.

## Results

### Body weight

**Fig 5:**
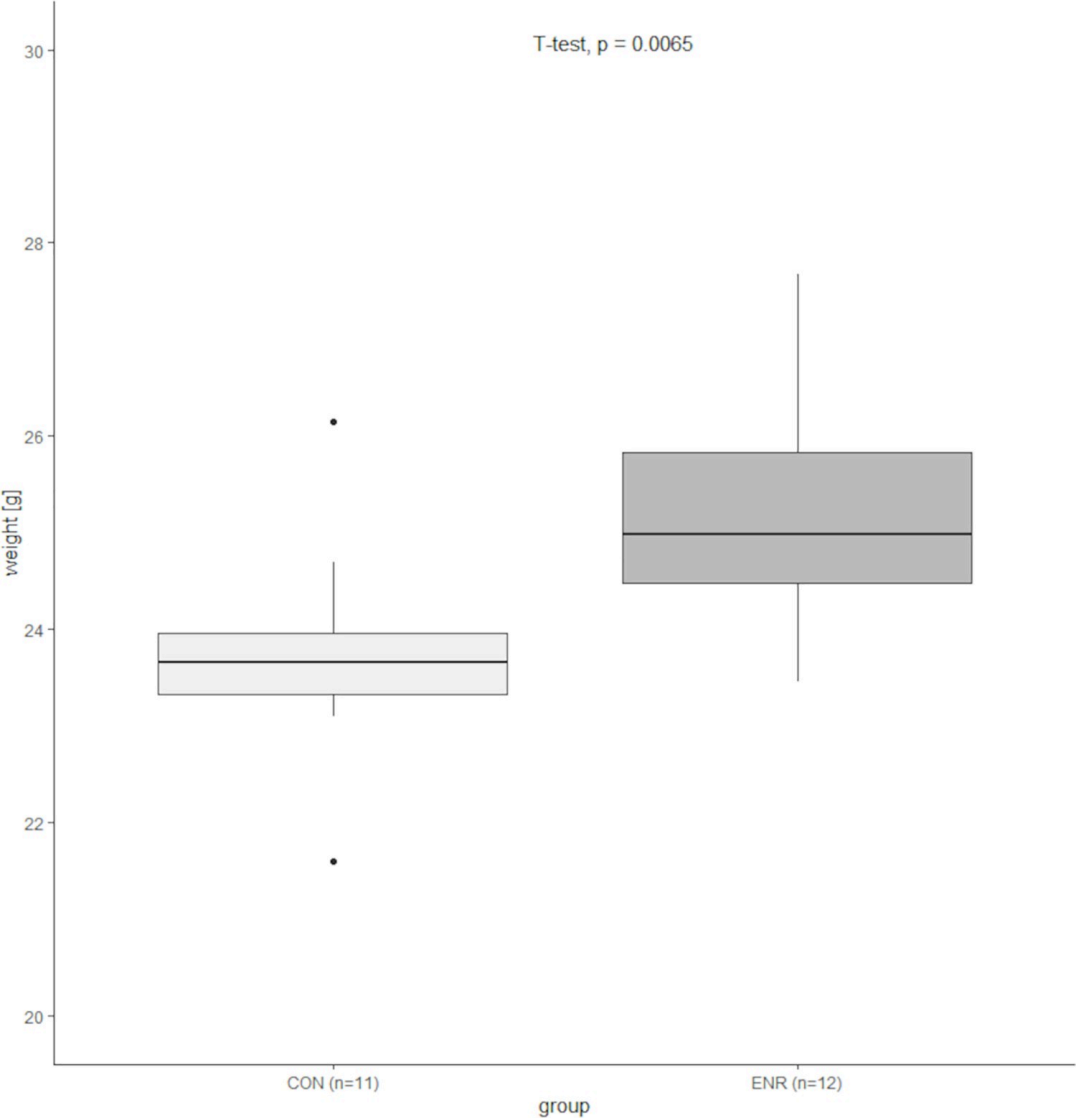
Body weight of enriched (n = 12) and conventional (n = 11) housed mice in [g]. Boxplots depict the median value (horizontal line), the interquartile range from 25th to 75th percentile of the data (box), and the minimum and maximum values lying above or below the box within 1.5 times the interquartile range (vertical lines), outliers (<q0.25–1.5 x IQR or > q0.75 + 1.5 x IQR) are drawn as filled circles.

The results of the body weight measurements of the conventional (n = 11) and enriched (n = 12) housed group are shown in Fig 5. Enriched housed mice (median 24.99 g) weighed significantly more (t-test, p = 0.0065) than their conventional (median 23.66 g) kept companions.

### Experiment 1: Observation of home cage behavior

**Fig 6:**
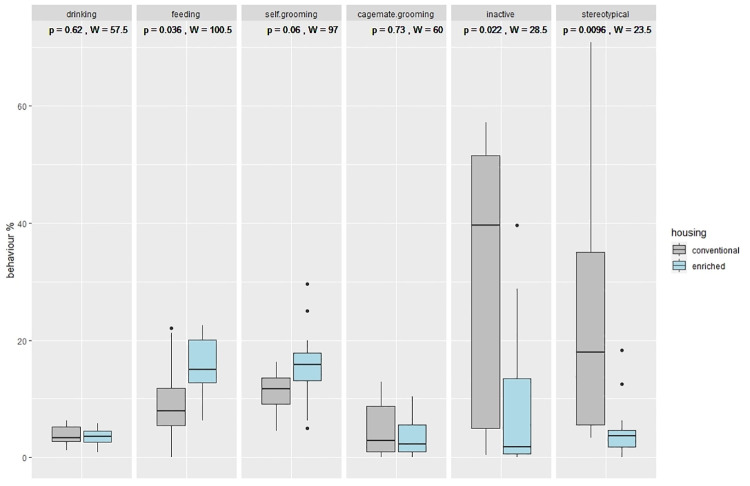
Observed behavior patterns of enriched (n = 12) and conventional housed (n = 11) mice in in % during the active phase. Boxplots depict the median value (horizontal line), the interquartile range from 25th to 75th percentile of the data (box), and the minimum and maximum values lying above or below the box within 1.5 times the interquartile range (vertical lines), outliers (<q0.25–1.5 x IQR or > q0.75 + 1.5 x IQR) are drawn as filled circles. The p-values of Wilcoxon-test results are above each boxplot. Stereotypical behavior includes scratching, wiping, bar orientated behavior, circling, jumping and route tracing.

The results of the compared behavioral observations are shown in Fig 6. Regarding the maintenance behaviors, no significant difference in occurrence could be shown neither in drinking (wilcoxon, p = 0.62) nor in self-grooming (wilcoxon, p = 0.06) between conventional (con) and enriched (enr) housed mice. Feeding behavior (wilcoxon, p = 0.036) was significantly increased in enriched housed mice. Active sociopositive behavior measured as cagemate-grooming (wilcoxon, p = 0.73) did not differ significantly between groups. Mice kept in conventional housing conditions showed significantly more inactive (wilcoxon, p = 0.022) as well as stereotypical (wilcoxon, p = 0.0096) behaviors compared with mice living in enriched housing. The following behavior patterns were summed up to stereotypical behavior: scratching, wiping, bar orientated behavior, circling, jumping, and route tracing. Raw data of all behaviors can be found in the (Table in S1 Table.)

### Inter rater reliability

To verify whether the direct observations were reliable according to our ethogram, a subset of the videos from 12 randomly selected mice (6 enriched, 6 conventional) was analyzed by a second observer. The agreement between both observers was 97.8% +/- 0.024 yielding a Cohen´s kappa of 0.96 which is representing a very high inter rater reliability [39]. There was no significant difference in the inter rater reliability between observations of mice from enriched compared with observations of mice living in conventional housing conditions (t-test, t = -0.93, df = 9.04, p = 0.38).

### Experiment 2: Observation of enrichment use

**Fig 7:**
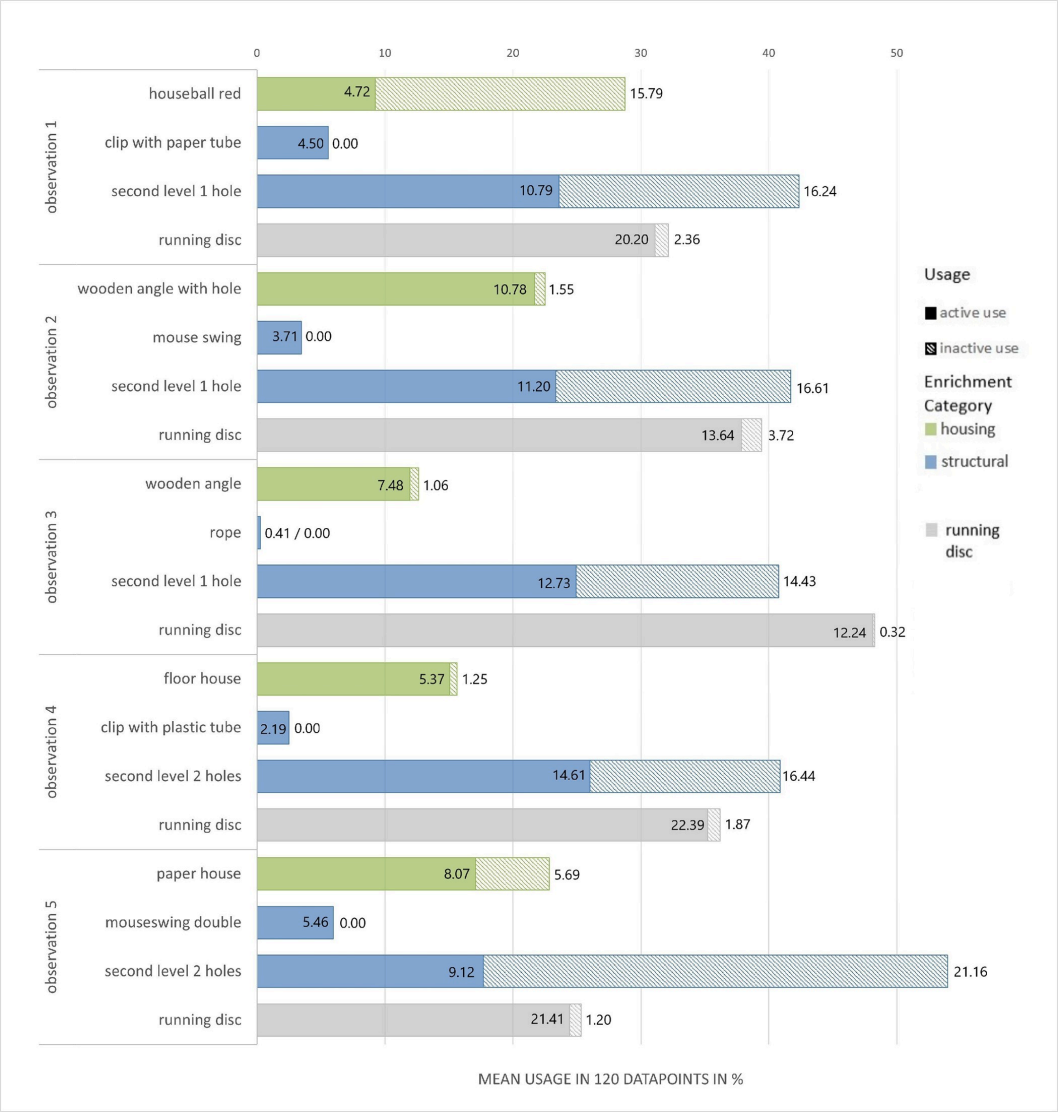
Video analysis of the active and inactive enrichment use in 5 observations of mice in enriched housing conditions (12 mice in 3 groups of 4 per cage) given as mean % of 120 datapoints. The running disc was provided in every enrichment item combination. Solid parts of the bars depict the active use of the respective elements and dashed parts of the bars depict the inactive use. Standard deviations for each stagged bar are given as numbers (inside bar for active use, outside for inactive use). Mice could use more than one item within each bout of 15s scan sampling therefore the added values of the bars might exceed 100.

**Fig 8:**
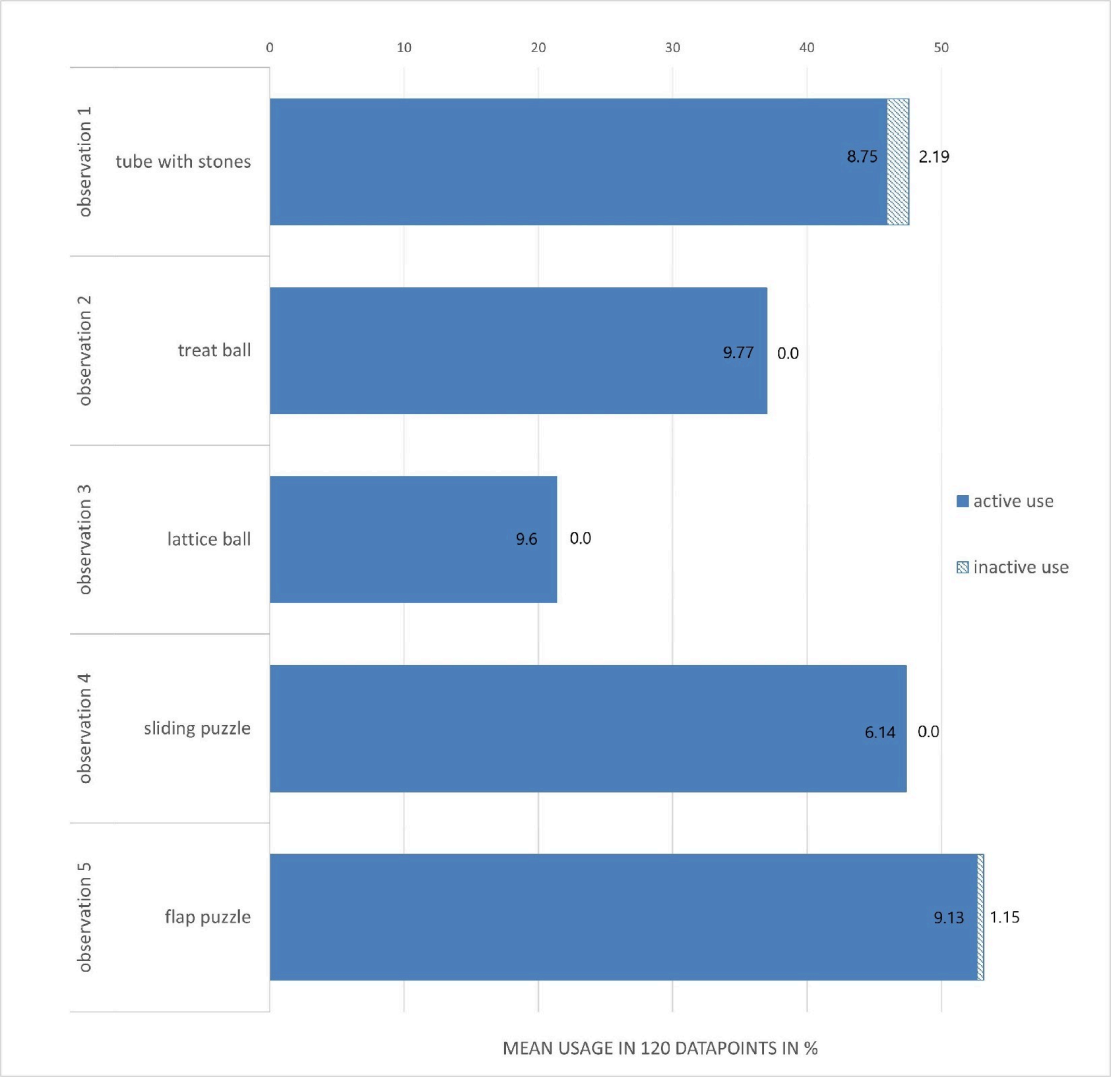
Video analysis of the active and inactive use of the foraging enrichments in 5 observations of mice in enriched housing conditions (12 mice in 3 groups of 4 per cage) given as mean % of 120 datapoints. Foraging enrichments were filled with millet during the observation period. Solid parts of the bars depict the active use of the respective elements and dashed parts of the bars depict the inactive use. Standard deviations for each stagged bar are given as numbers (inside bar for active use, outside for inactive use).

The results of the observation regarding the usage of the five enrichment setups are shown in Fig 7 and Fig 8 and as a table with the exact mean percentages in the (Table in S2 and S3 Tables). Fig 7 displays the use of ‘structural enrichment’, ‘housing enrichment’ and the running disc, Fig 8 shows the use of the ‘foraging enrichment’.

Within the category of ‘foraging enrichment’ the elements were used mostly actively. Mean active usage of foraging elements was lowest for the lattice ball (21.4%) and highest for the flap puzzle (52.6%). The treat ball, lattice ball, and sliding puzzle were not used inactively while the flap puzzle and tube with stones were used inactively at a low mean percentage (0.5% and 1.7%, respectively). From the category of ‘structural elements’, especially the second level with one or two holes was used often with mean active usage between 17.7% (second level 2 holes in observation 5) and 26.0% (second level 2 holes in observation 4) and mean inactive usage between 14.9% (second level 2 holes in observation 4) and 36.3% (second level 2 holes in observation 5). Overall, the second level was nearly used the same amount of time for active engagement as for resting purposes. All structural elements fixed at the top of the cage were the least used with mean active usage between 2.5% (clip with plastic tube) to 6% (mouseswing double). Because these could only be accessed through active behavior, inactive use is 0% for clip with paper tube, mouse swing, rope, clip with plastic tube, and mouseswing double. The mean active usage of the ‘housing enrichment’ was between 9.2% (houseball red) and 21.7% (wooden angle with hole). The inactive use of ‘housing enrichment’ was between 0.6% (floor house) and 19.5% (houseball red). As the running disc was always present, we analyzed its use separately as a category of its own. The running disc was highly used, mostly in an active way with mean active usage between 24.4% (observation [obs.] 5) and 48.1% (obs. 3), while nearly no inactive use occurred with mean inactive usage between 0.1% (obs. 3) and 1.6% (obs. 2).

The most frequently used items by all mice from each category were the flap puzzle from the ‘foraging enrichment’ category (active use: 52.6%, inactive use: 0.5%), the second level with 2 holes in observation setup 5 (active use: 17.7%, inactive use: 36.3%) from the ‘structural enrichment’ category, the houseball red (active use: 9.2%, inactive use: 19.5%) from the ‘housing enrichment’ category. The running disc was present in all observations and its mean active usage was 35.4% and the mean inactive use was 0.9%).

## Discussion

Within the context of refining animal experimentation, the goal of this study was to investigate the influence of an enriched environment on home cage behavior in mice and to assess the amount and kind of interaction with the presented enrichment items. It is already well known that shelters and nesting material are preferred by mice especially during the times when they rest [40]. Here we used focal animal sampling via video observations during the active phase of the mice for comparing behavior observed from enriched and conventional housed mice.

A second observation of the mice in enriched housing conditions within five sets of enrichment combinations served for analyzing the amount of either active or inactive interaction with the presented enrichment items.

Analyzing the influence of enrichment provision on home cage behavior revealed that drinking and cagemate-grooming were not influenced noticeably, whereas mice of the enriched housing conditions showed more self-grooming and more feeding behavior accompanied by heavier body weights. Due to the fact, that when comparing the rate of inactive behavior, mice in the conventional housing condition spent more time being inactive compared to the enriched housed group, it could be assumed that mice in enriched conditions may have higher caloric requirements as they show an increased interaction with their living environment and therefore display more feeding behavior. Studies examining the effect of an enriched environment on food intake remain inconclusive to date, as André et al. [20] found no difference in food intake and body weight when enriching mice with a shelter and nesting material, others showed mice in conventional conditions without nesting material to consume more food and weighing less [41]. These discrepancies may arise due to the different enrichment items used in the studies as providing mice with nesting material is known to facilitate body temperature control and therefore could reduce loss of energy expenditure for thermal regulation [42]. However, providing mice with a shelter for hiding and as a refuge may have smaller effects on food intake and body weight than on behavioral parameters. Thus, when assessing the effect of enrichment on feeding behavior, the characteristics of the enrichment offered should be considered. As our study is not aiming to analyze the effect of nesting material on behavior and thermoregulation, both groups received appropriate nesting material and the effect of increased feeding behavior is more likely to be induced by the higher amount of activity shown by enriched housed mice.

Inactivity, if increased under barren housing conditions, can also be a parameter indicative for decreased well-being in mice. Being inactive but awake was shown to reflect a sign of boredom in mink housed in non-enriched conditions compared to enriched housed animals [43]. Awake inactivity has been discussed as being a reaction to chronic stress as an alternative to stereotypical behavior in animals [44] and also correlated with depressive-like behaviors e.g., increased immobility in the forced swim test in mice [45]. Nip et al. [46] also found mice, in conventional keeping conditions compared to enriched kept mice, to be more agonistic and developed higher rates of stereotypical and inactive-but-awake behavior suggesting a negative impact of the barren housing condition on welfare in mice. In our study, the lack of objects for mice to interact with in conventional housing could also be considered a chronic stressor for the animals. Further analyses are needed to determine whether the inactive behavior of mice in conventional housing conditions may be a direct indicator of impaired well-being.

Another established sensitive parameter for animal welfare is the amount of stereotypical behavior shown [47]. Stereotypical behavior is defined as fixed repeated behavioral patterns lacking recognizable goal or function [47]. It has been shown that stereotypical behavior is associated with impoverished keeping conditions in many species including mice [47, 48]. In addition it has been shown in various studies that stereotypies can be reduced and prevented by enrichment [11, 12, 49, 50]. The results of our study confirm and extend these findings and thereby underline the need for a varied environment for laboratory mice. We argue that reducing the development of behavioral deficits like stereotypical behavior, is a profound basis for enabling the development of a normal behavioral repertoire as requested by the EU Directive 2010/63. Mice in enriched housing conditions displayed significantly less stereotypical behaviors including fixed repeated behavior patterns of route tracing, jumping, circling, bar-mouthing behavior, wiping and scratching. Some of these behavior patterns are thought to represent redirected escape attempts from the captive, less stimulating housing condition [51, 52]. In addition, stereotypical behavior may be an indicator of boredom in captive animals if other more specific causative factors like brain dysfunction [53] or specific frustrations i.e., inadequate diets or absence of appropriate nesting materials can be excluded [54]. Boredom, and animal boredom in particular, is an elusive topic that has received too little attention from the scientific community. Peter Toohey formulates predictability, monotony, and confinement as key criteria for the development of boredom [55]. Although this refers to human boredom, these factors also epitomize the typical life of captive (laboratory) animals. Indeed, preliminary studies suggest that laboratory animals can experience boredom that can significantly affect their well-being [54, 56]. Considering the numerous studies that have found a causal relationship between enriched housing conditions and improved cognitive capacity and neuroplasticity, the obvious inverse conclusion is that a low-stimulus environment could have devastating effects on these very processes. We emphasize here that chronic, inescapable boredom indeed could be a crucial factor in this.

With regard to concerns that enrichment may be a potential source for an increase in variability, our data showed the contrary: Most behavioral data did not differ in variability between mice from different housing conditions. However, a larger variability occurred in conventional housed mice for stereotypical behavior and for the time spent being inactive. This is in line with previous studies also showing no evidence that data from enriched housing was less reliable compared with data derived from conventional housing [20, 23, 57, 58]. Furthermore, laboratory animals in impoverished housing conditions may not be able to develop skills necessary for adequate performance in behavioral tests. This can consequently have a major impact on the validity of these tests, in addition to welfare implications.

The second observation of our study referred to the investigation of the utilization of the provided enrichment items. It is of note that views of what constitutes an enrichment item have changed over the years. In the past even providing nesting material to a barren cage was considered as enrichment and there is still no consensus definition of the term by now. We therefore classified the items by categorizing them according to their potential usefulness to the animals from an anthropomorphic position. These categories are "structural elements", "housing elements", and "foraging elements", and thus each serves different needs of the mice. As the mice got familiar with the enrichment elements and their use throughout the housing period and during observation within their home cage, there was no disturbance in possible group dynamics and the observations resemble their actual usage at the testing days, reflecting realistic laboratory conditions. It should be noted that the observation of animals kept in groups has a statistical limitation, as the use behavior within a cage unit may not be completely independent of the use of the same enrichment item by other cage mates. Since female mice are mostly socially living animals, this method for investigation of preferences for different enrichment items enabled to test the animals as a group [59] and maybe more applicable to the normal laboratory group housing situation [60].

Foraging enrichment elements were highly used in the 30 min observational time. As the favored item of this category, the flap puzzle with two reward holes covered by a flap on each side, was used the most during the observational time. We even observed all four mice of one group to be able to interact with it at once but did not quantify the amount of simultaneous interaction time. It is of note that the provision of treats as a classical reward is very common for companion animals. In laboratory animals treats are almost exclusively used in classical or operant conditioning as a means for increasing performance [5]. Although we did not directly measure how important the enrichment items were in terms of motivational strength, we observed that the animals showed high interest in solving the riddles of the foraging enrichment category. The motivation to interact with these items certainly was enhanced by providing millet seeds as a reward. Being able to engage in activities perceived as rewarding likely serves to achieve a more positive welfare state [4]. Overall, we highly recommend providing treats hidden in puzzle boxes as a novel form of cognitive stimulation and activity engaging enrichment.

Distinctive usage rates were observed for the enrichment elements assigned to the "structural" category. Elements fixed on the cage top were the least used elements in all observations independent of the other design elements presented and seemingly less interesting for the mice. It must be kept in mind that they also offer the smallest interaction surface and this could have negatively influenced the outcome of the use. On the other hand, the second level, either with one or two holes, was highly used in the observational period. This second level was used actively for climbing, gnawing or as a viewing platform as well as inactively as a hiding place and a place to groom or rest. Both active and inactive use were contributing similar amounts to total usage of the second level. As an additional observation, enriched housed mice exclusively used the space under the second level for nest building. Therefore, we recommend the use of those structural enrichment elements, which increase the usable space in the home cage.

The running disc was considered a separate category and was used frequently in the 30 minute video observation. Variability of the intensity and type of use between the different observations indicates an influence of the presence of other enrichment elements in the given constellation that may be more interesting to the animals. In addition, a running disc provides the animals with the opportunity for physical activity in their home cage. Mice in the wild are physically active and move extensively to explore, mate, defend, and search for food [61]. The use of running wheels by captive animals may also serve to compensate for differences in the input and output of energy, to keep temperature and/or metabolism at an acceptable level [62]. However, excessive running on a running wheel is also discussed to reflect a form of stereotypical behavior, addictive behavior, or even a laboratory artifact [63]. In contrast, our data shows that if mice have access to different enrichment elements at the same time, the use of the running disc is not the predominant behavior. In the same vein, a recent study supports the notion that in group housed mice no signs of stereotypical running wheel behavior were found [64]. Moreover, the known positive effects of exercise on cell proliferation and neurogenesis [65, 66], as well as on spatial learning and memory formation in mice [67–69] must be considered. Finally, the running discs we used in this study do not force the mice to run in a bent positions as in a small running wheel which has been discussed as a potential health and welfare concern due to ventral arching of the spine or an hyperflexion of the tail [63]. Overall, being physical active exerts beneficial effects on cognition, affect, and general health, and thus on animal welfare. Running wheels or discs offer the animals the opportunity to be physically active even in the very limited space of an conventional home cage and, if the experimental design of the study allows, should be considered in species-appropriate cage enrichment.

Housing elements are primarily used as resting or sleeping places. Since in this study the behavioral observation was carried out in the dark phase, i.e. in the active phase of the animals, only a limited statement can be made about the extent to which the housing elements provided were used as resting or sleeping places. Here work of van Loo [70] revealed that for resting during the inactive time mice preferred the Sheperd Shack (Shepherd Specialty Papers, Kalamazoo, Michigan US) over a triangular red plastic house (Tecniplast, Milan, Italy). Soerensen et al. [71] found strain differences in mice in the amount of usage of the triangular red plastic house as a resting place. Our work therefore shows that housing enrichment also suit well for interactive engagement, like climbing on/over or gnawing at. We believe that providing a shelter should be considered to be standard for contemporary housing of laboratory mice. The fact that housing enrichment items were also used in an active way might be a useful additional information when deciding which type of housing shall be provided for laboratory mice.

It has to be kept in mind for making general conclusions about the tested enrichment items that there are possible interaction effects between the items that we presented in parallel. In order to minimize this interaction, we chose an interval length of 15s, which was sufficient to allow usage of all items if the mice would have wanted.

Our analysis of inter rater reliability from Experiment 1 furthermore revealed a Cohen’s kappa value of .96 which corresponded to an almost perfect level of agreement according to McHugh [72] for our observational method. We attribute this high agreement to the one-zero sampling method using a reasonable time frame and a comprehensible ethogram. This method is therefore an easy-to-use approach to measure animal behavior and obtain reliable and reproducible results.

However, for a varied and stimulating housing environment, a simultaneous provision of different enrichment items is deemed necessary [73–75]. Our observational analysis of the interaction with the presented enrichment items represents one way of gaining knowledge about how differently categorized enrichment elements are perceived by mice. However, the anthropomorphic categorization of enrichment elements may not always reflect the actual interaction of mice with these elements. Nonetheless, our categorization may serve as an attempt to resolve the ongoing confusion caused by the use of "enrichment" as a generic term for any additional elements. Due to the elaborate method of manual evaluation of the videos, preference for different enrichment items could only be determined for a relatively short period of time and only during the active phase of the mice. Long-term studies using automated evaluation of preferences for different enrichment items over at least one circadian rhythm in mice [60] or the combined assessment of behavior and stay time [76], may provide further insights here. Combining automated tracking and behavioral analysis may be the most appropriate approach. Therefore, we would like to draw the attention to a related study in which we examined the preference of mice for enrichment items using multiple binary choice tests that were conducted over a 46-hour period [32].

## Conclusion

Our study corroborates the positive influence of a complex and enriched environment on the well-being of mice and underlines the importance of a diverse environment for healthy laboratory animals and thus for reliable animal models for biomedical research. Furthermore, systematic observation of the use of enrichment items in their home cages revealed pronounced preferences for specific enrichment items. There is widespread agreement that a stimulating living environment is vital to animal well-being. In order for laboratory animals to be able to perform their behavioral repertoire to the best of their ability, species-specific requirements must be implemented in the cage design.

## Supporting information

S1 File 3D printing template of the flap puzzle. (7Z)

S2 File 3D printing template of the treat ball. (7Z)

S1 Table Video analysis of the behaviors of 11 mice (conventional = 5, enriched = 6) in 240 datapoints during a 60-minute observational period displayed in percentage. (PDF)

S2 Table Video analysis of the mean active and inactive use of the enrichment items in 120 datapoints during a 30-minute observational period displayed in percentage from 5 observations in 12 mice. (JPG)

S3 Table Video analysis of the active and inactive use of the enrichment items in 120 datapoints during a 30-minute observational period displayed in percentage from 5 observations in 12 mice. (PDF)

S4 Table Permanently available items for the standard housed and enriched housed group. (PDF)

S5 Table Enrichment items used for the enriched housed mice during husbandry and experiments categorized by their intended type of use. (PDF)

S6 Table Ethogram for the analysis of the enrichment use of the enriched housed mice in experiment 2. (PDF)
