# Supplementary material for: Evaluation of different types of enrichment - their usage and effect on home cage behavior in female mice
Source: PLoS One. 2021 Dec 23;16(12):e0261876. doi: 10.1371/journal.pone.0261876 (PMC8699725; doi:10.1371/journal.pone.0261876)
Supplement: S1 Table — (PDF) [file pone.0261876.s003.pdf]

ID	housing	behaviour	percentage
391312	conventional	route_tracing	0.00
391542	conventional	route_tracing	0.00
391287	conventional	route_tracing	0.00
391380	conventional	route_tracing	66.67
381659	conventional	route_tracing	0.00
382209	conventional	route_tracing	0.00
391321	conventional	route_tracing	0.00
392223	conventional	route_tracing	0.00
382813	conventional	route_tracing	0.00
391404	conventional	route_tracing	0.83
kTr	conventional	route_tracing	0.00
392552	enriched	route_tracing	0.00
392543	enriched	route_tracing	0.00
392970	enriched	route_tracing	0.00
382754	enriched	route_tracing	0.00
382965	enriched	route_tracing	0.00
382748	enriched	route_tracing	0.00
392751	enriched	route_tracing	0.00
392701	enriched	route_tracing	0.00
383308	enriched	route_tracing	0.00
382179	enriched	route_tracing	0.00
391015	enriched	route_tracing	0.00
391487	enriched	route_tracing	0.00
391312	conventional	jumping	0.00
391542	conventional	jumping	0.00
391287	conventional	jumping	0.00
391380	conventional	jumping	1.67
381659	conventional	jumping	0.00
382209	conventional	jumping	0.42
391321	conventional	jumping	0.00
392223	conventional	jumping	0.00
382813	conventional	jumping	0.00
391404	conventional	jumping	24.58
kTr	conventional	jumping	0.00
392552	enriched	jumping	0.00
392543	enriched	jumping	0.00
392970	enriched	jumping	0.00
382754	enriched	jumping	0.00
382965	enriched	jumping	0.00
382748	enriched	jumping	0.00
392751	enriched	jumping	0.00
392701	enriched	jumping	0.00
383308	enriched	jumping	0.00
382179	enriched	jumping	0.00
391015	enriched	jumping	0.00
391487	enriched	jumping	0.00
391312	conventional	circling	5.42
391542	conventional	circling	1.25
391287	conventional	circling	33.33
391380	conventional	circling	0.00
381659	conventional	circling	0.00
382209	conventional	circling	13.33
391321	conventional	circling	1.25
392223	conventional	circling	5.42
382813	conventional	circling	0.83
391404	conventional	circling	0.42
kTr	conventional	circling	0.00
392552	enriched	circling	0.00
392543	enriched	circling	0.00
392970	enriched	circling	0.00
382754	enriched	circling	0.00
382965	enriched	circling	0.00
382748	enriched	circling	0.00
392751	enriched	circling	0.00
392701	enriched	circling	0.00
383308	enriched	circling	0.00
382179	enriched	circling	0.00
391015	enriched	circling	0.00
391487	enriched	circling	0.00
391312	conventional	bar_orientated_behaviour	16.25
391542	conventional	bar_orientated_behaviour	9.17
391287	conventional	bar_orientated_behaviour	5.83
391380	conventional	bar_orientated_behaviour	2.50
381659	conventional	bar_orientated_behaviour	4.17
382209	conventional	bar_orientated_behaviour	5.42
391321	conventional	bar_orientated_behaviour	2.08
392223	conventional	bar_orientated_behaviour	12.50
382813	conventional	bar_orientated_behaviour	6.25
391404	conventional	bar_orientated_behaviour	16.67
kTr	conventional	bar_orientated_behaviour	3.33
392552	enriched	bar_orientated_behaviour	2.08
392543	enriched	bar_orientated_behaviour	3.75
392970	enriched	bar_orientated_behaviour	0.83
382754	enriched	bar_orientated_behaviour	1.25
382965	enriched	bar_orientated_behaviour	16.25
382748	enriched	bar_orientated_behaviour	0.00
392751	enriched	bar_orientated_behaviour	12.50
392701	enriched	bar_orientated_behaviour	5.83
383308	enriched	bar_orientated_behaviour	4.17
382179	enriched	bar_orientated_behaviour	3.75
391015	enriched	bar_orientated_behaviour	3.33
391487	enriched	bar_orientated_behaviour	3.75
391312	conventional	wiping	0.00
391542	conventional	wiping	0.00
391287	conventional	wiping	0.00
391380	conventional	wiping	0.00
381659	conventional	wiping	0.00
382209	conventional	wiping	0.00
391321	conventional	wiping	0.00
392223	conventional	wiping	0.00
382813	conventional	wiping	0.00
391404	conventional	wiping	0.00
kTr	conventional	wiping	0.00
392552	enriched	wiping	0.00
392543	enriched	wiping	0.00
392970	enriched	wiping	0.00
382754	enriched	wiping	0.00
382965	enriched	wiping	0.00
382748	enriched	wiping	0.00
392751	enriched	wiping	0.00
392701	enriched	wiping	0.00
383308	enriched	wiping	0.00
382179	enriched	wiping	0.00
391015	enriched	wiping	0.00
391487	enriched	wiping	0.00
391312	conventional	scratching	7.92
391542	conventional	scratching	0.00
391287	conventional	scratching	1.25
391380	conventional	scratching	0.00
381659	conventional	scratching	0.00
382209	conventional	scratching	0.00
391321	conventional	scratching	0.00
392223	conventional	scratching	0.00
382813	conventional	scratching	0.00
391404	conventional	scratching	2.92
kTr	conventional	scratching	0.00
392552	enriched	scratching	0.00
392543	enriched	scratching	0.00
392970	enriched	scratching	0.00
382754	enriched	scratching	0.00
382965	enriched	scratching	2.08
382748	enriched	scratching	0.00
392751	enriched	scratching	0.00
392701	enriched	scratching	0.42
383308	enriched	scratching	0.00
382179	enriched	scratching	0.00
391015	enriched	scratching	0.00
391487	enriched	scratching	0.00
391312	conventional	inactive	0.83
391542	conventional	inactive	26.25
391287	conventional	inactive	2.92
391380	conventional	inactive	0.42
381659	conventional	inactive	52.92
382209	conventional	inactive	45.42
391321	conventional	inactive	54.17
392223	conventional	inactive	39.58
382813	conventional	inactive	57.08
391404	conventional	inactive	7.08
kTr	conventional	inactive	50.00
392552	enriched	inactive	0.83
392543	enriched	inactive	0.00
392970	enriched	inactive	0.83
382754	enriched	inactive	0.83
382965	enriched	inactive	28.75
382748	enriched	inactive	39.58
392751	enriched	inactive	0.00
392701	enriched	inactive	18.75
383308	enriched	inactive	2.92
382179	enriched	inactive	3.33
391015	enriched	inactive	11.67
391487	enriched	inactive	0.00
391312	conventional	drinking	5.83
391542	conventional	drinking	5.42
391287	conventional	drinking	2.92
391380	conventional	drinking	5.00
381659	conventional	drinking	1.25
382209	conventional	drinking	2.50
391321	conventional	drinking	2.50
392223	conventional	drinking	3.33
382813	conventional	drinking	3.33
391404	conventional	drinking	6.25
kTr	conventional	drinking	4.17
392552	enriched	drinking	5.42
392543	enriched	drinking	5.83
392970	enriched	drinking	4.58
382754	enriched	drinking	4.17
382965	enriched	drinking	3.33
382748	enriched	drinking	0.83
392751	enriched	drinking	4.58
392701	enriched	drinking	1.67
383308	enriched	drinking	2.92
382179	enriched	drinking	2.92
391015	enriched	drinking	0.83
391487	enriched	drinking	3.75
391312	conventional	feeding	22.08
391542	conventional	feeding	7.92
391287	conventional	feeding	4.17
391380	conventional	feeding	6.67
381659	conventional	feeding	8.75
382209	conventional	feeding	0.00
391321	conventional	feeding	6.67
392223	conventional	feeding	10.00
382813	conventional	feeding	2.08
391404	conventional	feeding	13.75
kTr	conventional	feeding	21.25
392552	enriched	feeding	9.58
392543	enriched	feeding	21.25
392970	enriched	feeding	22.50
382754	enriched	feeding	15.83
382965	enriched	feeding	6.25
382748	enriched	feeding	18.33
392751	enriched	feeding	20.00
392701	enriched	feeding	13.33
383308	enriched	feeding	14.17
382179	enriched	feeding	20.42
391015	enriched	feeding	10.83
391487	enriched	feeding	14.17
391312	conventional	self.grooming	13.75
391542	conventional	self.grooming	9.58
391287	conventional	self.grooming	10.42
391380	conventional	self.grooming	15.00
381659	conventional	self.grooming	11.67
382209	conventional	self.grooming	8.75
391321	conventional	self.grooming	4.58
392223	conventional	self.grooming	13.33
382813	conventional	self.grooming	16.25
391404	conventional	self.grooming	12.08
kTr	conventional	self.grooming	7.92
392552	enriched	self.grooming	20.00
392543	enriched	self.grooming	6.25
392970	enriched	self.grooming	25.00
382754	enriched	self.grooming	15.00
382965	enriched	self.grooming	7.50
382748	enriched	self.grooming	5.00
392751	enriched	self.grooming	16.25
392701	enriched	self.grooming	17.08
383308	enriched	self.grooming	15.00
382179	enriched	self.grooming	29.58
391015	enriched	self.grooming	15.42
391487	enriched	self.grooming	16.25
391312	conventional	cagemate.grooming	1.25
391542	conventional	cagemate.grooming	1.25
391287	conventional	cagemate.grooming	0.00
391380	conventional	cagemate.grooming	0.00
381659	conventional	cagemate.grooming	12.92
382209	conventional	cagemate.grooming	11.67
391321	conventional	cagemate.grooming	5.00
392223	conventional	cagemate.grooming	7.50
382813	conventional	cagemate.grooming	0.83
391404	conventional	cagemate.grooming	2.92
kTr	conventional	cagemate.grooming	10.00
392552	enriched	cagemate.grooming	5.00
392543	enriched	cagemate.grooming	0.00
392970	enriched	cagemate.grooming	7.50
382754	enriched	cagemate.grooming	2.08
382965	enriched	cagemate.grooming	0.00
382748	enriched	cagemate.grooming	2.08
392751	enriched	cagemate.grooming	0.00
392701	enriched	cagemate.grooming	1.25
383308	enriched	cagemate.grooming	4.17
382179	enriched	cagemate.grooming	10.42
391015	enriched	cagemate.grooming	2.50
391487	enriched	cagemate.grooming	10.42
