# Supplementary figures and images for: Evaluation of different types of enrichment - their usage and effect on home cage behavior in female mice
Source: PLoS One. 2021 Dec 23;16(12):e0261876. doi: 10.1371/journal.pone.0261876 (PMC8699725; doi:10.1371/journal.pone.0261876)

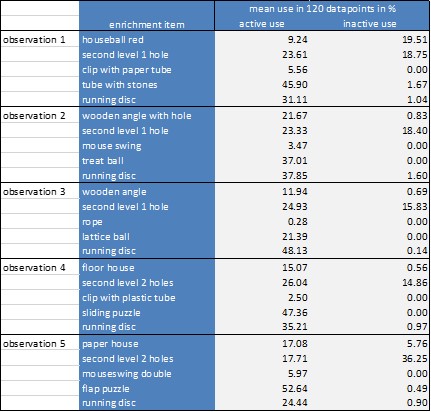

Supplement: S2 Table — (JPG) [file pone.0261876.s004.jpg]
