# Supplementary material for: Evaluation of different types of enrichment - their usage and effect on home cage behavior in female mice
Source: PLoS One. 2021 Dec 23;16(12):e0261876. doi: 10.1371/journal.pone.0261876 (PMC8699725; doi:10.1371/journal.pone.0261876)
Supplement: S3 Table — (PDF) [file pone.0261876.s005.pdf]

mouse	observation 1									
	houseball red		second level 1 hole		clip with papertube		running disc		tube with stones	
	active	inactive	active	inactive	active	inactive	active	inactive	active	inactive
1	18.3333333	14.1666667	20.8333333	18.3333333	4.16666667	0	20	0	40.8333333	0
2	10.8333333	0.83333333	15	3.33333333	1.66666667	0	60.8333333	0.83333333	35.8333333	0.83333333
3	9.16666667	9.16666667	28.3333333	24.1666667	15	0	33.3333333	0.83333333	53.3333333	4.16666667
4	10.8333333	0	25.8333333	0	5	0	70.8333333	0	42.5	0
5	6.66666667	55	27.5	2.5	2.5	0	11.6666667	0	58.3333333	2.5
6	10.8333333	15.8333333	31.6666667	19.1666667	10	0	10.8333333	0	52.5	1.66666667
7	15	7.5	49.1666667	12.5	5.83333333	0	15	0.83333333	38.3333333	5
8	5.83333333	23.3333333	15.8333333	25.8333333	3.33333333	0	20	8.33333333	62.5	5.83333333
9	11.6666667	30	27.5	20	12.5	0	35.8333333	1.66666667	40	0
10	6.66666667	35.8333333	18.3333333	15.8333333	2.5	0	42.5	0	40	0
11	2.5	29.1666667	16.6666667	20.8333333	2.5	0	42.5	0	47.5	0
12	2.5	13.3333333	6.66666667	62.5	1.66666667	0	10	0	39.1666667	0

mouse	observation 2									
	wooden angle with hole		second level 1 hole		mouseswing		running disc		treatball	
	active	inactive	active	inactive	active	inactive	active	inactive	active	inactive
1	14.1666667	5	15	26.6666667	0.83333333	0	37.5	0	38.3333333	0
2	19.1666667	0	12.5	14.1666667	0	0	52.5	0	40	0
3	15.8333333	0	23.3333333	8.33333333	2.5	0	40.8333333	0	48.3333333	0
4	26.6666667	0	20	25.8333333	0	0	25.8333333	0	45.8333333	0
5	8.33333333	0	15	47.5	2.5	0	23.3333333	0	50	0
6	24.1666667	0	40	5	5	0	47.5	5	31.6666667	0
7	30	1.66666667	35.8333333	16.6666667	8.33333333	0	40.8333333	0.83333333	31.6666667	0
8	4.16666667	0	2.5	50	0.83333333	0	12.5	12.5	47.5	0
9	35	0.83333333	25	1.66666667	4.16666667	0	40	0.83333333	20.8333333	0
10	39.1666667	0	25.8333333	4.16666667	5.83333333	0	42.5	0	32.5	0
11	14.1666667	2.5	38.3333333	1.66666667	0	0	62.5	0	35	0
12	29.1666667	0	26.6666667	19.1666667	11.6666667	0	28.3333333	0	22.5	0

mouse	observation 3									
	wooden angle		second level 1 hole		rope		running disc		lattice ball	
	active	inactive	active	inactive	active	inactive	active	inactive	active	inactive
1	7.5	0	11.6666667	50.8333333	0	0	21.6666667	0	26.6666667	0
2	7.5	0	11.6666667	7.5	0	0	34.1666667	0	20	0
3	18.3333333	3.33333333	29.1666667	15	0.83333333	0	54.1666667	0	27.5	0
4	13.3333333	1.66666667	21.6666667	10.8333333	0	0	60	0	31.6666667	0
5	4.16666667	0	5.83333333	14.1666667	0.83333333	0	36.6666667	0.83333333	9.16666667	0
6	5	0	30.8333333	0.83333333	0	0	49.1666667	0	10.8333333	0
7	10.8333333	0.83333333	21.6666667	7.5	0.83333333	0	48.3333333	0	5.83333333	0
8	0.83333333	0	15	15	0	0	51.6666667	0	15.8333333	0
9	19.1666667	1.66666667	50	12.5	0	0	58.3333333	0	35.8333333	0
10	12.5	0.83333333	30	39.1666667	0.83333333	0	43.3333333	0.83333333	30.8333333	0
11	17.5	0	35.8333333	6.66666667	0	0	56.6666667	0	23.3333333	0
12	26.6666667	0	35.8333333	10	0	0	63.3333333	0	19.1666667	0

mouse	observation 4									
	floorhouse		second level 2 holes		clip with plastictube		running disc		sliding puzzle	
	active	inactive	active	inactive	active	inactive	active	inactive	active	inactive
1	15.8333333	0	16.6666667	5	1.66666667	0	56.6666667	6.66666667	37.5	0
2	16.6666667	0	33.3333333	0	7.5	0	72.5	0	43.3333333	0
3	10	0	15.8333333	44.1666667	1.66666667	0	15	0.83333333	51.6666667	0
4	7.5	0	9.16666667	17.5	0	0	55.8333333	0	48.3333333	0
5	18.3333333	0	31.6666667	0.83333333	2.5	0	60.8333333	0	46.6666667	0
6	11.6666667	0	22.5	0.83333333	3.33333333	0	52.5	0	60	0
7	7.5	0	9.16666667	0	0	0	12.5	0	50	0
8	25	0	11.6666667	5	0	0	26.6666667	0.83333333	48.3333333	0
9	20.8333333	1.66666667	54.1666667	18.3333333	4.16666667	0	18.3333333	1.66666667	41.6666667	0
10	19.1666667	4.16666667	37.5	29.1666667	4.16666667	0	13.3333333	0.83333333	40	0
11	13.3333333	0.83333333	25.8333333	44.1666667	3.33333333	0	20.8333333	0	52.5	0
12	15	0	45	13.3333333	1.66666667	0	17.5	0.83333333	48.3333333	0

mouse	observation 5									
	paper house		second level 2 holes		mouseswing double		running disc		flap puzzle	
	active	inactive	active	inactive	active	inactive	active	inactive	active	inactive
1	28.3333333	9.16666667	27.5	38.3333333	9.16666667	0	32.5	0	56.6666667	0
2	12.5	7.5	17.5	9.16666667	3.33333333	0	56.6666667	0	48.3333333	0
3	27.5	0	29.1666667	20	14.1666667	0	40	0.83333333	59.1666667	0
4	21.6666667	1.66666667	33.3333333	6.66666667	17.5	0	47.5	0.83333333	57.5	0
5	7.5	5	14.1666667	14.1666667	5	0	53.3333333	0	42.5	0
6	11.6666667	0	18.3333333	64.1666667	1.66666667	0	9.16666667	0	38.3333333	0
7	25.8333333	0	12.5	24.1666667	4.16666667	0	32.5	0	42.5	3.33333333
8	20.8333333	13.3333333	5	62.5	0	0	5	2.5	43.3333333	0
9	3.33333333	3.33333333	8.33333333	57.5	0.83333333	0	1.66666667	2.5	63.3333333	0
10	15	10.8333333	15	45	4.16666667	0	4.16666667	0	59.1666667	2.5
11	19.1666667	16.6666667	24.1666667	36.6666667	9.16666667	0	5.83333333	3.33333333	65	0
12	11.6666667	1.66666667	7.5	56.6666667	2.5	0	5	0.83333333	55.8333333	0
