# Supplementary material for: Evaluation of different types of enrichment - their usage and effect on home cage behavior in female mice
Source: PLoS One. 2021 Dec 23;16(12):e0261876. doi: 10.1371/journal.pone.0261876 (PMC8699725; doi:10.1371/journal.pone.0261876)
Supplement: S4 Table — (PDF) [file pone.0261876.s006.pdf]

deployment	enrichment item	
**standard house for the standard housed mice**	**triangular house** (mouse house, TECNIPLAST®)	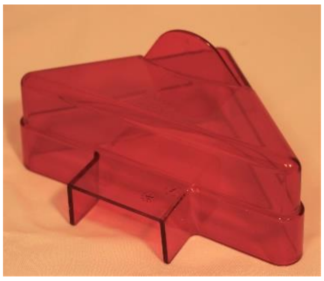
**standard house in video observation experiment**	**running wheel** (fast-trac + mouse igloo, Bio-Serv®)	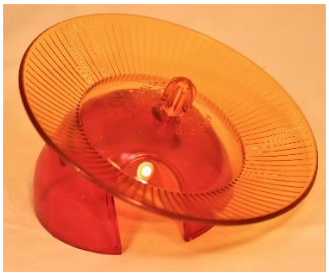
**permanently available**	**paper nesting** (cellusose paper unbleached 20x20 cm, Lohmann & Rauscher International GmbH & CO KG)	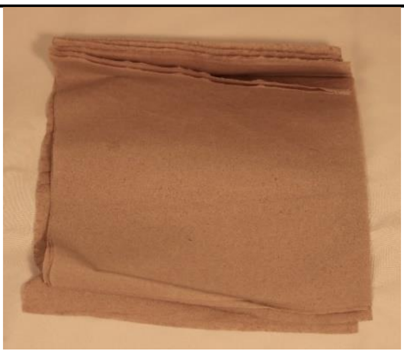
	**cotton roll** (dental cotton roll size 3, MED-COMFORT)	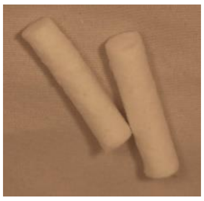
