# Supplementary material for: Evaluation of different types of enrichment - their usage and effect on home cage behavior in female mice
Source: PLoS One. 2021 Dec 23;16(12):e0261876. doi: 10.1371/journal.pone.0261876 (PMC8699725; doi:10.1371/journal.pone.0261876)
Supplement: S5 Table — (PDF) [file pone.0261876.s007.pdf]

category	enrichment item	
nesting	**fine wood wool** (H0234-NBF, ABEDD®)	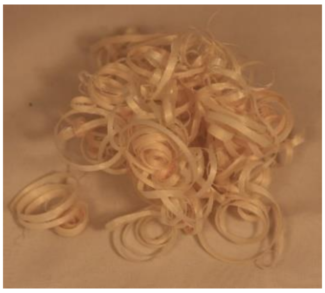
	**coarse wood wool** (H0234-NBU, ABEDD®)	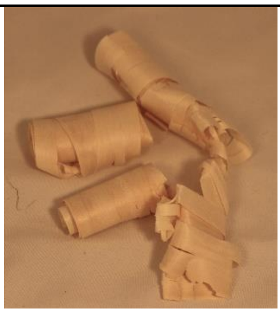
	**square hemp pads** (H3279-10 eco- hemp, ssniff Spezialdiäten GmbH)	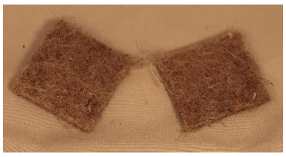
	**folded paper strips** (sizzlenest®, datesand Ltd)	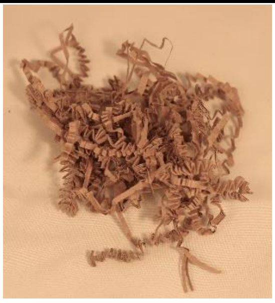
	**mid coarse wood wool** (NBGE012, ABEDD®)	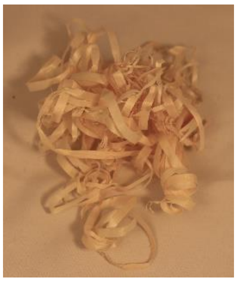

**housing**	**house ball**<br>(crawlball, Bio-Serv®)	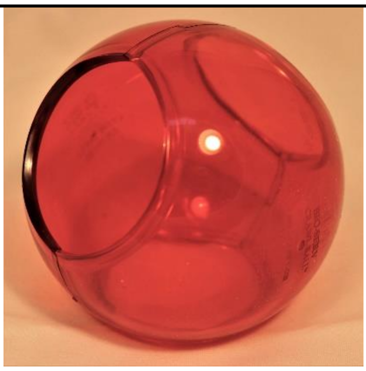
	**floor house**<br>(safe harbor, Bio-Serv®)	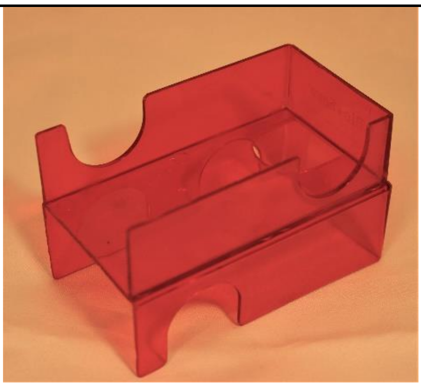
	**paper house**<br>(LBS Serving Biotechnology)	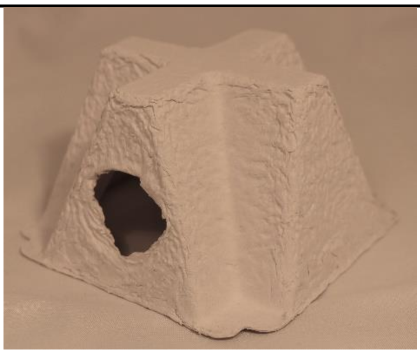
	**wooden angle**<br>(climbing roof, ABEDD®)	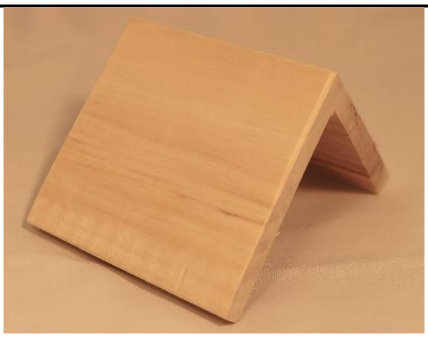
	**holed wooden angle**<br>(holed climbing roof, ABEDD®)	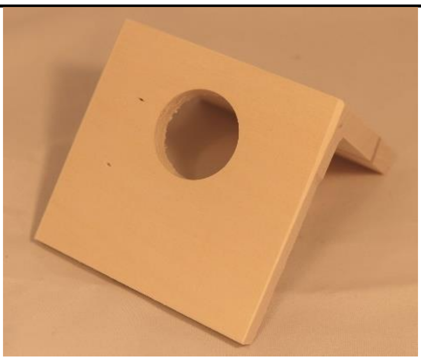

	**second level, 1 hole** (1 hole lying boards for cage type III, ABEDD®)	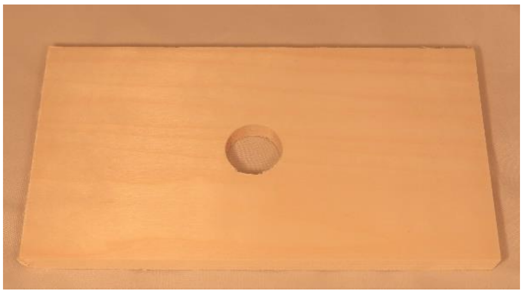
	**second level, 2 holes** (2 hole lying boards for cage type III, ABEDD®)	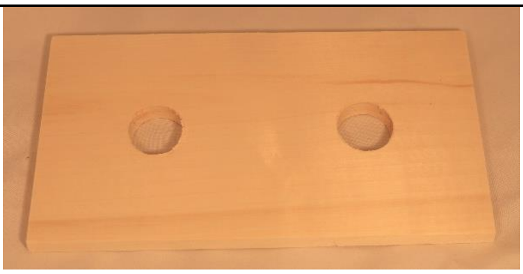
**structural**	**clip with paper tube** (38 x 1.25 x 75 mm play tunnel and tunnel clip, Datesand Ltd)	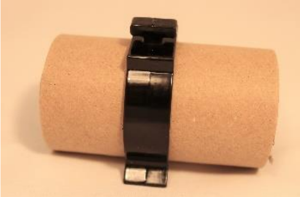
	**clip with plastic tube** (Plexiglas tube transparent 70mm Ø, KUS and tunnel clip, Datesand Ltd)	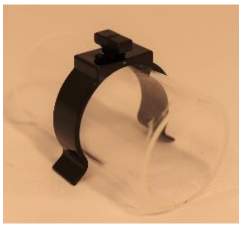
	**mouse swing** (single mouse swing, Datesand Ltd)	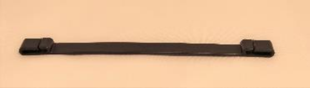
	**mouse swing double** (double mouse swing, Datesand Ltd)	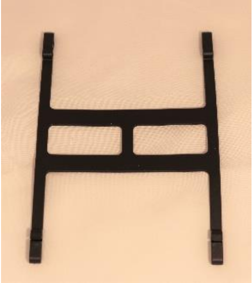

	**rope**<br>(jute yarn 6-ply, 6mm, Rayher 4200531)	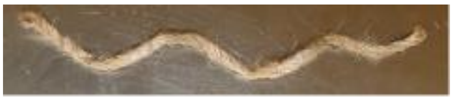
**active**	**treat ball**<br>(self-designed and printed with PLA filament, Ultimaker extended 3)	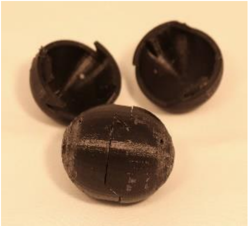
	**sliding puzzle**<br>(Interactive Smart Toy , Living World® green)	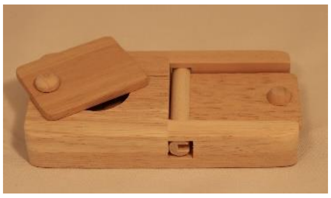
	**tube with stones**<br>(mouse tunnel, Bio-Serv® and white marble pebbles 15 – 25 mm Ø, Min2C Natural Minerals)	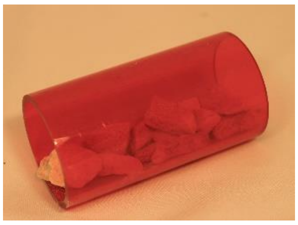
	**lattice ball with ball chain**<br>(Hol-ee Roller® size mini, JW®)	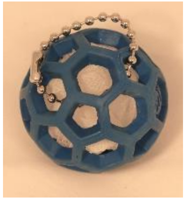
	**flap puzzle**<br>(self-designed and printed with PLA filament, Ultimaker extended 3)	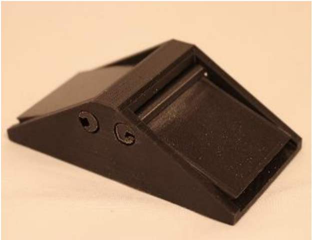
