# Supplementary material for: Evaluation of different types of enrichment - their usage and effect on home cage behavior in female mice
Source: PLoS One. 2021 Dec 23;16(12):e0261876. doi: 10.1371/journal.pone.0261876 (PMC8699725; doi:10.1371/journal.pone.0261876)
Supplement: S6 Table — (PDF) [file pone.0261876.s008.pdf]

enrichment item	active use	inactive use
**house ball**	The mouse sits on the house ball, runs through the house ball, climbs over it or gnaws at it.	The mouse sits in the house ball, sleeps in it or grooms itself/other in it.
**floor house**	The mouse sits on the floor house, runs through the floor house, climbs over it or gnaws at it.	The mouse sits in the floor house, sleeps in it or grooms itself/other in it.
**wooden angle**	The mouse sits on the wooden angle, runs through the wooden angle, climbs over it or gnaws at it.	The mouse sits underneath the wooden angle, sleeps in it or grooms itself/other in it.
**holed wooden angle**	The mouse sits on the holed wooden angle, runs through the holed wooden angle, climbs over it or gnaws at it.	The mouse sits underneath the holed wooden angle, sleeps in it or grooms itself/other in it.
**paper house**	The mouse sits on the paper house, runs through the paper house, climbs over it or gnaws at it.	The mouse sits underneath the paper house, sleeps in it or grooms itself/other in it.
**second level, 1 hole**	The mouse sits on the second level, climbs over it or gnaws at it.	The mouse sits under the second level, sleeps under it or grooms itself/other under it.
**second level, 2 holes**	The mouse sits on the second level, climbs over it or gnaws at it.	The mouse sits under the second level, sleeps under it or grooms itself/other under it.
**clip + paper tube**	The mouse sits in the cardboard tube, climbs through/over it or gnaws at it.	The mouse sleeps in the cardboard tube or grooms itself/other in it.
**clip + plastic tube**	The mouse sits in the plastic tube, climbs through/over it or gnaws at it.	The mouse sleeps in the plastic tube or grooms itself/other in it.
**mouse swing**	The mouse sits in the mouse swing, climbs through/over it or gnaws at it.	The mouse sleeps in the mouse swing or grooms itself/other in it.
**mouse swing double**	The mouse sits in the mouse swing double, climbs through/over it or gnaws at it.	The mouse sleeps in the mouse swing double or grooms itself/other in it.
**rope**	The mouse climbs the rope or gnaws at it.	-
**treat ball**	The mouse moves the ball with its muzzle/paws or sniffs at it.	The mouse sits/sleeps on or with direct body contact to the treat ball.
**sliding puzzle**	The mouse moves the sliding or hinged lid of the sliding puzzle with the nose or paws or gnaws or sniffs at it.	The mouse sleeps on the sliding puzzle.

**tube + stones**	The mouse manipulates the stones with its paws/nose, runs over/through the tube or sits inside on the stones and eats millet.	The mouse rests or sleeps in/on the tube.
**lattice ball**	The mouse climbs or gnaws at the lattice ball or stretches from the ground towards the grid ball in order to gnaw at it or take-out contents and eat them underneath the ball (millet).	- (no inactive use available)
**flap puzzle**	The mouse moves a flap lid of the puzzle with the nose/paws or gnaws or sniffs at the flap puzzle.	The mouse sleeps on the flap puzzle.
**running wheel**	The mouse climbs on/over the running wheel, runs on the running table or runs through the house of the running wheel.	The mouse sits in the house of the running wheel, sleeps in it or grooms itself/other in it.
